# Correlates of sex trading among male non-injecting drug users in Myanmar: a cross-sectional study

**DOI:** 10.1186/s12954-016-0123-0

**Published:** 2016-12-05

**Authors:** Yu Mon Saw, Thu Nandar Saw, Kyi Mar Wai, Krishna C. Poudel, Hla Hla Win

**Affiliations:** 1 Department of Healthcare Administration, Graduate School of Medicine, Nagoya University, 65 Tsurumai-cho, Showa-ku, Nagoya, 466-8550 Japan; 2 Nagoya University Asian Satellite Campuses Institute, Nagoya, Japan; 3 Myanma Perfect Research, Yangon, Myanmar; 4 Department of Community and Global Health, Graduate School of Medicine, the University of Tokyo, Tokyo, Japan; 5 Department of Human Ecology, Graduate School of Medicine, the University of Tokyo, Tokyo, Japan; 6 Department of Health Promotion and Policy, School of Public Health and Health Sciences, University of Massachusetts Amherst, Amherst, MA USA; 7 Department of Preventive and Social Medicine, University of Medicine 1, Yangon, Myanmar

**Keywords:** Exchange sex, Sex trading, Drug users, Myanmar

## Abstract

**Background:**

Sex trading is a recognized risk factor for human immune deficiency virus infection and other sexually transmitted infections among non-injecting drug users (NIDUs). However, very little research has addressed the factors associated with sex trading among male NIDUs in Myanmar.

**Methods:**

A cross-sectional study was conducted from January to February 2010 using the respondent-driven sampling method. In total, 210 NIDUs aged between 18 and 49 years, with no history of injecting drug use, and who used non-injected illicit drugs in the last 6 months were recruited. Face-to-face interviews were conducted using a structured questionnaire to collect information on participants’ sexual and drug use behaviors. Binary and multivariate logistic regressions were applied to analyze the resulting data.

**Results:**

Of 210 NIDUs, 84 (40%) reported involvement in the sex trade during the last 3 months. In the adjusted model, factors associated with sex trade involvement included homosexual preference (adjusted odds ratio [AOR] 4.90; 95% confidence interval [CI] 1.61–14.95), having more than two partners (*AOR* = 3.88; 95% CI 1.55–9.72), had a regular job (*AOR* = 5.10; 95% CI 1.65–15.72), use of stimulant drugs rather than opiate (*AOR* = 2.38; 95% CI 1.10–5.15), and who used drugs more than twice per day.

**Conclusions:**

More than one third of NIDUs were involved in sex trading. This study suggested that further comprehensive intervention programs that aim to reduce risk factors of trading sex among NIDUs may consider including NIDUs who used stimulant drugs, had regular/full-time jobs, used drugs more than twice per day, and had homosexual preferences.

## Background

Sex trading, for money or drugs, has emerged as a significant public health concern due to a wide range of negative health impacts [1]. Among these, sexually transmitted infections (STIs), including HIV, are one of the recognized risk factors among drug users [2]. In addition, the confounding effects of drug use on psychological distress may contribute to correlations between sex trading and psychological status such as depression, suicide attempts, and social and physical victimization among drug-using populations [1, 3].

The risk of STIs/HIV infection may still be present among non-injecting drug users (NIDUs) through high-risk sexual behaviors even though they do not inject drug intravenously [4, 5]. For example, in theUnited States of America, HIV prevalence among injecting drug users (IDUs) is nearly identical to that among NIDUs due to high-risk sexual behaviors [6]. Many studies reported that sex trading was associated with non or inconsistent use of condoms [7–10] homelessness, unemployment, less education, drug use, and heterosexual (bisexual) behavior [11]. In addition, sex trading is associated with higher rates of adult sexual victimization, experience of violence, and previous history of being sexually abused in childhood [7].

Myanmar stands as the second largest illicit opium producer in Asia, and the drug-using population may be a key population at risk [12]. The United Nations Office on Drugs and Crime estimated 300,000 drug users in 2008. In Myanmar, approximately 212,000 people were living with HIV in 2014. The HIV epidemic largely effects IDUs, men who had sex with men, and female sex workers [13]. HIV prevalence among IDUs was 23.1%, while adult prevalence was 0.54% in 2014 [14]. However, HIV prevalence among NIDUs is unavailable.

Researchers and policy makers have been paying more attention to IDUs due to their risky injecting behaviors compared to NIDUs [6, 15, 16]. In addition, while several researchers are giving attention to sex trading among male IDUs [11, 17], only a paucity of research addresses factors associated with sex trading among male NIDUs, especially in the developing country settings. This study aims to identify the risk factors of sex trading among male NIDUs in Lashio, Myanmar. The findings of this study may help in fine-tuning suitable national strategies in Myanmar and in Southeast Asia in general to find possible solutions for the health problems as consequences of sex trading.

## Methods

### Study design and participants

The cross-sectional study was conducted from January to February 2010 in Lashio city, Northern Shan State. In total, 210 male NIDUs were recruited using a respondent-driven sampling (RDS) method. The inclusion criteria of this study were as follows: (1) aged 18 years or older, (2) with no history of injecting drug use, (3) had used illicit non-injecting drugs in the previous 6 months, (4) not suffering from a serious drug dependency, and (5) able to speak the Myanmar language.

Participants were recruited using an RDS method that included coupon and dual incentives system [5, 18]. The details of the sampling procedure are presented elsewhere [19]. The first respondent was recruited from a local drop-in center as a seed, and that participant was requested to introduce three drug users through a coupon system. All the participants were requested to recruit their friends/partners within the 14 days before the coupon expiry date. For each interview, participants received 2000 Kyats (US$ 2.5) and an information, education, and communication pack that included two condoms with gel packs as a primary incentive. They were eligible to receive the secondary incentives of 500 kyats (0.5$) only after the NIDUs whom they recruited participated in the study.

### Measures and data collection

The data was collected using a standardized, precoded questionnaire adapted from several different studies that had been implemented in Myanmar. Sexual behaviors questions were adapted from the Behavioral Surveillance Survey (BSS) Questionnaire of the Ministry of Health, Myanmar and the sociodemographic characteristics, drug use behavior, and health services utilization questions were adapted from the Rapid Assessment and Response on Drug Use and from the HIV Survey of the Asian Harm Reduction Network, Myanmar [19, 20]. The questionnaire was translated from English to the Myanmar language and was pretested among a sample of NIDUs residing outside the study sites in November, 2009 by the researchers. Back-translation of the instrument from the Myanmar language to English was done before and after the pretest to ensure semantic equivalence via a pilot study for assessing content validity, appropriateness, and question comprehensibility. The questionnaire was then revised as necessary. Training was given to the data collectors for two days on how to conduct the interview, content of the questionnaire, data quality, and ways to approach respondents [19].

The dependent variable was ever-traded sex for money or drugs in the past 3 months. Sex trading was defined as a participant having exchanged sex for money or drugs as measured by the following question: “In the past three month, did you trade/exchange sex for money or drugs (i.e. received money or drugs through sex-trading)?” [21]. The response of the above question was dichotomized as ever-traded sex (1 = yes) versus never traded sex (0 = no, do not know, and no response). The independent variables included sociodemographic characteristics such as age, ethnicity, marital status, education, employment status, and living status; drug use-related variables such as the most used drug type, history of poly drug use, frequency of drug use, and age; sexual behaviors related characteristics such as age of sexual initiation, sexual orientation, number of sexual partner, and consistent condom use; and HIV testing behaviors, and health service utilization related characteristics such as ever tested HIV and STI/HIV diagnosis [19, 22]. The interview was conducted where NIDU participants felt comfortable discussing this issue, such as a local drop-in center, shooting galleries, or the participant’s home. Each interview took 30 to 45 min.

### Data analysis

Data were double entered and cross-checked using Microsoft Excel, and analyzed using the Statistical Package for the Social Sciences version 18 (SPSS Inc., Chicago, IL, USA). The descriptive statistics were calculated for the sociodemographic, drug use, and sexual behavior characteristics of study participants. In all the analyses, the level of significance was set at *P* < 0.05 (two-tailed). Logistic regression models were fitted to the data to model the crude associations between the background characteristics of the participants and the involvement in sex trading. Finally, all the covariates were simultaneously entered into the stepwise regression analysis to see the most significant variables.

### Ethical considerations

This study was approved by the Research Ethics Committee of the Graduate School of Medicine, the University of Tokyo, Japan and the Institutional Ethical Review Committee, Department of Medical Research (Lower Myanmar), Ministry of Health, Yangon, Myanmar. The objectives of the study were made clear to the respondents before their voluntary participation, and individual written informed consent was obtained from all the participants. Each participant was allowed to withdraw from the study at any time. Confidentiality of the entire data set was maintained at all stages of the data collection and analyses.

## Results

### Descriptive statistics

In total, 210 NIDUs, 31.4% belonged to the Shan ethnic group and more than half of them (55.2%) were below the age 25 years (Table 1). The majority of the respondents reported being single (52.4%), and the reminder reported as follows: 32.4% married and 15.2% divorced/widower. Regarding their educational status, 39.0% reported having a high school or above degree, 83.8% had regular/full-time job, and approximately one third of them (31.9%) had migrated from other areas.

**Table 1:** Sociodemographic characteristics of participants (*N* = 210)

Characteristics	Number of cases (*N*)	Percentage (%)
Age		
<25	116	55.2
≥25	94	44.8
Ethnicity		
Burma	45	21.4
Shan	66	31.4
Kachin	48	22.9
Others	51	24.3
Marital status		
Single	110	52.4
Married	68	32.4
Divorced/widower	32	15.2
Education		
Primary/no formal education	61	29.1
Secondary education	67	31.9
High school or above	82	39.0
Employment status		
Non-regular job	34	16.2
Regular/full-time job	176	83.8
Living status		
Migrant	67	31.9
Resident	143	68.1

### Correlates of sex trading: crude analysis

In the unadjusted analyses, NIDUs who had regular/full-time job, had lifetime history of poly drug use, and used drug more than twice per day were more likely to report sex trading in the past 3 months (Table 2). Participants, who used stimulant drug in the past 3 months (unadjusted odds ratio [UOR] 3.90, 95% confidence interval [CI] 2.15–7.09) and who initiated sexual intercourse before the age of 16 years, were more likely to report sex trading (Table 2).

**Table 2:** Bivariate analysis for correlates of sex trading among non-injecting drug users

Characteristics	Number	%	UOR	95% CI
Age				
<25	116	55.2		
≥25	94	44.8	0.89	(0.50–1.53)
Ethnicity				
Burma	45	21.4		
Shan	66	31.4	0.89	(0.41–1.92)
Kachin	48	22.9	0.98	(0.43–2.23)
Others	51	24.3	0.81	(0.36–1.85)
Marital status				
Single	110	52.4		
Married	68	32.4	0.99	(0.53–1.83)
Divorced/widower	32	15.2	1.03	(0.46–2.29)
Education				
Primary/no formal education	61	29.1		
Secondary education	67	31.9	0.66	(0.33–1.32)
High school or above	82	39.0	0.54	(0.27–1.06)
Employment status				
Non-regular job	34	16.2		
Regular/full-time job	176	83.8	4.94	(2.11–18.49)^b^
Living status				
Migrant	67	31.9		
Resident	143	68.1	0.63	(0.35–1.13)
Most used drug type in the past 3 months				
Heroine/opium	98	46.7		
Stimulant	112	53.3	3.90	(2.15–7.09)^a^
Had history of poly drug use				
Never	58	27.6		
Ever	152	72.4	2.38	(1.22–4.65)^c^
Frequency of drug use per day				
≤2	69	32.9		
>2	141	67.1	4.96	(2.45–10.03)^a^
Age of sexual initiation				
≤16	123	58.6		
>16	87	41.4	0.33	(0.18–0.61)^a^
Had used drugs before or during sex in the past 3 months				
No	45	21.4		
Yes	165	78.6	2.85	(1.32–6.13)^b^
Sexual Orientation				
Heterosexual	76	36.2		
Bisexual	55	26.2	3.65	(1.58–8.45)^b^
Homosexual	79	37.6	11.38	(5.16–25.08)^a^
Number of sexual partner in the past 3 months				
≤2	64	30.5		
>2	164	69.5	7.60	(3.39–17.06)^a^
Had new casual partner in the last 1 month				
No	55	26.2		
Yes	155	73.8	5.65	(2.51–12.74)^a^
Consistent condom use in the past 3 months				
No	138	65.7		
Yes	72	34.3	0.65	(0.36–1.18)
Ever test HIV				
No	114	54.3		
Yes	96	45.7	0.89	(0.51–1.56)
Ever received drug treatment				
No	139	66.2		
Yes	71	33.8	0.56	(0.31–1.02)
Diagnosed with an STI/HIV in the past 3 months				
No	92	43.8		
Yes	118	56.2	4.71	(2.53–8.77)^a^
Ever been in prison or jail				
No	189	90.0		
Yes	21	10.0	1.25	(0.60–2.57)
Perceived HIV risk				
No	120	57.1		
Yes	90	42.9	1.63	(0.93–2.84)
Ever registered as a drug user				
No	173	82.4		
Yes	37	17.6	1.18	(0.57–2.41)

*UOR* unadjusted odds ratio, *CI* confidence interval

Here a, b, and c indicates *p* < 0.001, *p* < 0.01, and *p* < 0.05

Significantly higher odds ratio of trading sex was observed among participants who had homosexual preferences (UOR 11.38; 95% CI 5.16–25.08) or bisexual preferences (UOR 3.65; 95% CI 1.58–8.45; Table 2). Participants who had more than two partners (UOR 4.96; 95% CI 2.45–10.03) in the past 3 months were more likely to be involved in sex trading. NIDUs, who reported that they had casual partners in the last 1 month were 5.65 times (95% CI 2.51–12.74) times more likely to report having trade sex. In addition, a significant association was observed between sex trading and those who had been diagnosed with a STIs/HIV in the past 6 months (UOR 4.71; 95% CI 2.53–8.77; Table 2).

### Correlates of sex trading: multivariate analysis

In the stepwise regression analysis, having a regular job (adjusted odds ratio [AOR] 5.10; 95% confidence interval [CI] 1.65–15.72) was found to be positively associated with sex trading (Table 3). Having more than two partners (*AOR* = 3.88; 95% CI 1.55–9.72), having homosexual preferences (*AOR* = 4.90; 95% CI 1.61–14.95), stimulant drug use (*AOR* = 2.38; 95% CI 1.10–5.15), and frequency of drug use more than twice per day (*AOR* = 2.62; 95% CI 1.19–5.77) also remained significantly associated with sex trading within 3 months. In addition, a significant association was observed between sex trading and drug use before and during sex in the past 3 months (*AOR* 2.76; 95% CI 1.08–7.03).

**Table 3:** Multivariate analysis for correlates of sex trading among non-injecting drug users

Characteristics	AOR	95% CI
Employment status		
Non-regular job		
Regular/full-time job	5.10	(1.65–15.72)^b^
Most used drug type in the past 3 months		
Heroine/opium		
Stimulant	2.38	(1.10–5.15)^c^
Frequency of drug use per day		
≤2		
>2	2.62	(1.19–5.77)^c^
Sexual orientation		
Heterosexual		
Bisexual	1.69	(0.55–5.18)
Homosexual	4.90	(1.61–14.95)^b^
Number of sexual partner in the past 3 months		
≤2		
>2	3.88	(1.55–9.72)^b^
Had used drugs before or during sex in the past 3 months		
No		
Yes	2.76	(1.08–7.03)^c^

*AOR* adjusted odds ratio, *CI* confidence interval

Here a, b, and c indicates *p* < 0.001, *p* < 0.01, and *p* < 0.05

## Discussion

This is the first study of the relationship between sex trading and associated risk factors among NIDUs in Myanmar. Findings from this study indicate that 40% of the NIDUs engaged in sex trading within the last 3 months. This extremely high prevalence rate confirms that sex trading is an alarmingly commonplace in this impoverished Southeast Asian nation that can potentially place NIDUs at high risk for HIV and other sexually transmitted diseases. The findings of this study document that the higher prevalence of sex trading among NIDUs is similar to the risk level of injection drug users carried out in different developing and developed nations [11, 23–25].

Our findings indicate that stimulant drug use is associated with involvement in sex trading among our sample. This is in line with previous observations that stimulant users often engage in sex trading and high-risk sexual behaviors with multiple sex partners [26–28]. Evidence also shows that some drug users traded sex for drugs, rather than money, to suppress the effects of drug withdrawal [27, 29, 30]. Among the NIDU population, stimulant drugs are believed to be a key contributor of engagement in high-risk sexual behavior [31–33]. These findings, therefore, suggest that preventive interventions addressing sex trading among NIDUs must target stimulant drug users.

Having multiple partners over a short period of time is believed to be a major behavioral factor of STIs [34]. Our findings revealed that participants who had more than two partners in the past 3 months were associated with sex trading. This finding is consistent with previous research, which showed that having multiple sexual partners was associated with increased high-risk sexual behaviors and may increase the risk of HIV and other STIs [35–37]. Another new important finding is that NIDUs whose frequency of drug use was higher (more than twice per day) were more likely to be involved in sex trading. This result is expected because as the frequency of drug use increases so does the likelihood of selling sex to procure drugs.

It has been shown that men who have sex with men were more likely to report a history of trading sex for money or drugs than were heterosexual men [38]. The present results also support the hypothesis indicating that NIDUs with homosexual preferences were more likely to report involvement in sex trading. Heavy substance use is highly prevalent in this community [39] and, along with poverty and economic needs (“survival sex”), it may be driving much of the ongoing HIV risk behaviors through the exchange of sex for money or drugs [40]. Moreover, an additional analysis was run in this study to examine this hypothesis, and we found that NIDUs with homosexual preferences have higher frequency of drug use (more than twice per day).

This study also shows that NIDUs who used drugs before and during sex in the past 3 months were positively associated with sex trading. This result is in accordance with the previous findings suggesting that using drugs and alcohol before or during sex leads an individual to engage in high-risk sexual behaviors [41–43].

Contrary to the previous studies, our findings demonstrated that sex trading was associated with having a regular income to live on or having a full-time job at the time of survey among male NIDUs in this study. Previous studies revealed that sex trading was associated with unemployment [10] and homelessness [11]. This may be explained by the fact that regular job holders may sometimes engage in sex trading for stimulant drugs even if they have a full-time job due to illicit drug accessibility, rather than money.

Some limitations should be considered when interpreting our findings. First, the data were based on the participants’ self-report. Regarding questions related to sexual behaviors, there is a possibility of under-reporting and social desirability bias related to the use of the face-to-face interviewing technique. However, in order to minimize this problem, peers of NIDUs were hired as interviewers to make participants feel more comfortable in reporting the truth. Also, confidentiality of the participants was carefully maintained throughout, with RDS identification codes used in place of the participants’ names. Second, there is a possibility of recall bias because participants were asked to report their behaviors in the past 3 months or more. However, to minimize the recall bias, we inquired about details of participants’ sexual behaviors and used validated and reliable instruments to collect data.

Finally, the current analyses are cross-sectional, and thus do not allow us to assess temporal relationships between variables or ascertainment of causal sequences. Despite such limitations, our findings have important implications with regard to designing interventions to reduce sex trade among the NIDUs in Myanmar and comparable settings. The conclusions drawn may also be useful for policy makers to refine and develop future sexual risk reduction programs for the NIDUs in Myanmar.

## Conclusions

In conclusion, the NIDUs in Myanmar engaged in trade sex at an elevated rate. That may be one of the reasons behind the higher prevalence of STIs/HIV infection in these populations. Our study recommends an effective comprehensive intervention to reduce risk factors of sex trade among the NIDUs, particularly focusing on NIDUs who used stimulant drugs, who used drugs heavily, had regular jobs, had homosexual preferences, and having multiple types of partners. Developing strategies that include the above interventions may play a crucial role in limiting the spread of HIV among NIDUs and their partners. Since these behaviors have major implications for HIV acquisition and public health, prevention efforts and targeted provision of addiction treatment to this population should be expanded.

## Abbreviations

AOR: Adjusted odds ratio
CI: Confidence intervals
HIV: Human immune deficiency virus
IDUs: Injecting drug users
NIDUs: Non-injecting drug users
RDS: Respondent-driven sampling
STIs: Sexually transmitted infections
UOR: Unadjusted odds ratio.
